# Supplementary material for: ACE2 polymorphisms as potential players in COVID-19 outcome
Source: PLoS One. 2020 Dec 28;15(12):e0243887. doi: 10.1371/journal.pone.0243887 (PMC7769452; doi:10.1371/journal.pone.0243887)
Supplement: S1 Table — RS: Reference SNP; Ref: Reference allele; Alt: Alternative allele; EUR: European; EAS: East Asian; SAS: South Asian; AMR: Ad Mixed American; AFR: African; NAM: Native American; BAP: Brazilian Admixed Population; ABM: Online Archive of Brazilian Mutations; GLO: Global (1000 Genomes). Bold exons are contained in common isoforms. (DOCX) [file pone.0243887.s001.docx]

| **S1 Table. The minor allele frequency (MAF) of SNPs in exonic regions of ACE2 that showed differences between the investigated populations.** | | | | | | | | | |
| --- | --- | --- | --- | --- | --- | --- | --- | --- | --- |
| **RS** | **EUR** | **EAS** | **SAS** | **AMR** | **AFR** | **NAM** | **BAP** | **ABM** | **GLO** |
| **rs147464721** | 0 | 0 | 0 | 0.002 | 0.005 | 0 | 0.014 | 0.002 | 0.002 |
| **rs889263894** | 0 | 0 | 0 | 0 | 0 | 0.034 | 0 | 0 | 0 |
| **rs1027571965** | 0 | 0 | 0 | 0 | 0 | 0.095 | 0 | 0 | 0 |
| **rs4646179** | 0 | 0 | 0 | 0.021 | 0.074 | 0 | 0.007 | 0.023 | 0.023 |
| **rs35803318** | 0.054 | 0 | 0 | 0.071 | 0.001 | 0.121 | 0.074 | 0.047 | 0.021 |
| **rs41303171** | 0.018 | 0 | 0.001 | 0.004 | 0 | 0 | 0 | 0.009 | 0.005 |
| **rs147311723** | 0 | 0 | 0 | 0.002 | 0.017 | 0 | 0 | 0.002 | 0.005 |
| **rs142017934** | 0 | 0 | 0 | 0 | 0.013 | 0 | 0 | 0 | 0.003 |
| **rs182366225** | 0 | 0.018 | 0 | 0 | 0 | 0 | 0 | 0 | 0.004 |
| rs16979941 | 0.003 | 0 | 0.082 | 0.038 | 0.378 | ND | ND | ND | 0.122 |
| ss1553623767 | 0.001 | 0 | 0.081 | 0.025 | 0.253 | ND | ND | ND | 0.086 |
| rs777900815 | 0 | 0 | 0.011 | 0 | 0 | ND | ND | ND | 0.002 |
| rs779675389 | 0 | 0 | 0 | 0 | 0.003 | ND | ND | ND | 0.001 |
| rs142660243 | 0.001 | 0.061 | 0.007 | 0.042 | 0.001 | ND | ND | ND | 0.02 |
| rs187539494 | 0.003 | 0 | 0.018 | 0.004 | 0 | ND | ND | ND | 0.005 |
| rs7890520 | 0.003 | 0 | 0.11 | 0.038 | 0.315 | ND | ND | ND | 0.11 |
| rs908004 | 0.389 | 0.424 | 0.242 | 0.46 | 0.818 | ND | ND | ND | 0.492 |
| rs3827466 | 0.35 | 0.017 | 0.293 | 0.218 | 0.034 | ND | ND | ND | 0.169 |
| RS: Reference SNP; Ref: reference allele; Alt: alternative allele; EUR: European; EAS: East Asian; SAS: South Asian; AMR: Ad Mixed American; AFR: African; NAM: Native American; BAP: Brazilian Admixed Population; ABM: Online Archive of Brazilian Mutations; GLO: Global (1000 Genomes). Bold exons are contained in common isoforms. | | | | | | | | | |
