# Supplementary material for: ACE2 polymorphisms as potential players in COVID-19 outcome
Source: PLoS One. 2020 Dec 28;15(12):e0243887. doi: 10.1371/journal.pone.0243887 (PMC7769452; doi:10.1371/journal.pone.0243887)
Supplement: S2 Table — *Insertion of an Alu mobile element relative to the reference. (DOCX) [file pone.0243887.s002.docx]

| **S2 Table. Chromosome position, reference (RS) and minor allele of SNPs (MAF>0.01) in ten thousand base pairs 5´ upstream of ACE2.** | | | | | | | |
| --- | --- | --- | --- | --- | --- | --- | --- |
| **Position** | **RS** | **Minor Allele** |  |  | **Position** | **RS** | **Minor Allele** |
| 15629748 | rs16997094 | C |  |  | 15625357 | rs150147953 | T |
| 15629452 | rs182288080 | G |  |  | 15624931 | rs140820246 | G |
| 15629447 | rs200235505 | CACTT |  |  | 15624522 | rs150974643 | A |
| 15629441 | rs2097723 | C |  |  | 15624419 | rs146962767 | C |
| 15629436 | rs766474171 | G |  |  | 15623942 | rs111719883 | A |
| 15629433 | rs200599510 | A |  |  | 15623378 | rs12009805 | C |
| 15629313 | rs199688052 | T |  |  | 15623374 | rs112312217 | C |
| 15629300 | rs191594254 | A |  |  | 15623017 | rs16997089 | G |
| 15629232 | rs149071726 | G |  |  | 15622377 | rs111515287 | G |
| 15628839 | rs144934578 | T |  |  | 15622242 | rs16997083 | T |
| 15628770 | rs145061276 | A |  |  | 15622077 | rs142049267 | G |
| 15628729 | rs185719886 | A |  |  | 15621988 | rs184697926 | C |
| 15628712 | rs778840079 | C |  |  | 15621888 | rs756087769 | T |
| 15628575 | rs139469582 | A |  |  | 15621879 | rs112593415 | G |
| 15628284 | rs185248824 | C |  |  | 15621777 | rs9698150 | C |
| 15627583 | rs111471604 | A |  |  | 15621753 | rs9698134 | T |
| 15627401 | rs139946832 | A |  |  | 15621721 | rs765471058 | T |
| 15627354 | rs112062094 | A |  |  | 15621720 | rs760084155 | A |
| 15627202 | rs34816996 | C |  |  | 15621700 | rs11336754 | ATT |
| 15626865 | . | *INS:ALU |  |  | 15621700 | rs11336754 | AT |
| 15626495 | rs199829224 | A |  |  | 15621700 | rs11336754 | A |
| 15626344 | rs141945167 | G |  |  | 15621565 | rs112621533 | C |
| 15626226 | rs778186530 | G |  |  | 15621257 | rs7885856 | A |
| 15626167 | rs113539251 | A |  |  | 15620852 | rs113009615 | T |
| 15626039 | rs113653961 | C |  |  | 15620464 | rs138035717 | A |
| 15625983 | rs5934250 | T |  |  | 15620340 | rs190509934 | C |
| 15625979 | rs138813533 | A |  |  | 15620301 | rs140394675 | G |
| 15625699 | rs144943713 | T |  |  | 15620300 | rs147718775 | G |
| 15625374 | rs73635826 | T |  |  | - | - | - |
| *Insertion of an Alu mobile element relative to the reference | | | | | | | |
