# Supplementary material for: ACE2 polymorphisms as potential players in COVID-19 outcome
Source: PLoS One. 2020 Dec 28;15(12):e0243887. doi: 10.1371/journal.pone.0243887 (PMC7769452; doi:10.1371/journal.pone.0243887)
Supplement: S3 Table — Population*: Population with greater MAF; RS: Reference SNP; Site: genic region; MAF*: minor allele frequency of population*; Subpopulation alert (MAF): Subpopulation with greater MAF; Others (MAF): MAF of the other populations (or populations without MAF = null); Modification (score): Type of molecular consequence and PolyPhen Score [32]; Hypothetical influence: based on the most probable hypothetical biological influence in disease; Men risk: based on hemizygous genotypic frequency (q); Women risk: based on homozygous genotypic frequency (q2); M/W: risk ratio between sexes to be a carrier of the minor allele only. (DOCX) [file pone.0243887.s003.docx]

| **S3 Table. ACE2 polymorphisms with the potential to influence in the structure or gene expression, and their population and sex differences.** | | | | | | | | | | |
| --- | --- | --- | --- | --- | --- | --- | --- | --- | --- | --- |
| **Population*** | **RS** | **Site** | **MAF*** | **Subpopulation alert (MAF)** | **Others (MAF)** | **Modification (score)** | **Hipothetical influence** | **Men risk** | **Women risk** | **M/W** |
| BAP | rs147464721 | codon 351 | 0.014 |  | absent | Synonymous | Neutral | 1.4% | 0.0% | 71.4 |
| ABM, AMR, AFR | rs4646179 | codon 690 | 0.074 | Nigerians (0.12) | absent | Synonymous | Neutral | 7.4% | 0.5% | 13.5 |
| NAM | rs35803318 | codon 749 | 0.121 | Peruvians and Spaniards (0.100) | EAS, SAS, AFR (null) | Synonymous | Neutral | 12.1% | 1.5% | 8.3 |
| EUR | rs41303171 | codon 720 | 0.018 | British (0.03) | absent | Asn - > Asp (0.02) | Unknown | 1.8% | 0.0% | 55.6 |
| AFR | rs147311723 | codon 731 | 0.017 | Nigerians (0.043) | EAS, SAS, AMR, ABM (null) | Leu -> Phe (0.941) | Unknown | 1.7% | 0.0% | 58.8 |
| NAM | rs1027571965 | codon 673 | 0.095 |  | absent | Ala -> Gly (0.045) | Unknown | 9.5% | 0.9% | 10.5 |
| NAM | rs889263894 | codon 541 | 0.034 |  | absent | Lys -> Ile (0.958) | Unknown | 3.4% | 0.1% | 29.4 |
| EAS | rs182366225 | 3´UTR | 0.018 | Vietnamese and Chinese (0.032) | absent | Upregulation | Worse | 1.8% | 0.0% | 55.6 |
| AFR | rs142017934 | 3´UTR | 0.013 | Nigerians and AFR in Barbados (0.026) | absent | Upregulation | Worse | 1.3% | 0.0% | 76.9 |
| NAM | rs2285666 | intron | 0.71 |  | EAS (0.54) to AFR (0.17) | Upregulation (Brain, Nerve) | Worse | 71.0% | 50.4% | 1.4 |
| AFR | rs4646140 | intron | 0.13 | Nigerians (0.17) | SAS (0.085) to NAM (0) | No evidence | May neutral | 13.0% | 1.7% | 7.7 |
| EAS | rs2097723 | Upstream | 0.42 |  | AMR (0.32) to AFR (0.07) | Upregulation (Brain/Nerve) | Worse | 42.0% | 17.6% | 2.4 |
| EUR | rs5934250 | Upstream | 0.47 |  | AMR (0.29) to EAS (0.01) | Downregulation (Brain, Nerve, Artery, Pituitary, Protate) | Better | 47.0% | 22.1% | 2.1 |

Population*: Population with greater MAF; RS: Reference SNP; Site: genic region; MAF*: minor allele frequency of population*; Subpopulation alert (MAF): Subpopulation with greater MAF; Others (MAF): MAF of the other populations (or populations without MAF=null); Modification (score): Type of molecular consequence and PolyPhen Score [32]; Hypothetical influence: based on the most probable hypothetical biological influence in disease; Men risk: based on hemizygous genotypic frequency (q); Women risk: based on homozygous genotypic frequency (q^2^); M/W: risk ratio between sexes to be a carrier of the minor allele only.
