# Supplementary material for: ACE2 polymorphisms as potential players in COVID-19 outcome
Source: PLoS One. 2020 Dec 28;15(12):e0243887. doi: 10.1371/journal.pone.0243887 (PMC7769452; doi:10.1371/journal.pone.0243887)
Supplement: S5 Table — (DOCX) [file pone.0243887.s005.docx]

**S4 Table. Tribe names and geographic coordinates of the Brazilian Amazon indigenous populations enrolled in this study.**

| **Tribe** | **Latitude** | **Longitude** |
| --- | --- | --- |
| Asurini do Koatinemo | -4.25584 | -52.2765 |
| Arara/Arara do Iriri | -3.74619 | -53.0491 |
| Araweté | -4.92912 | -52.4775 |
| Asurini do Trocará | -3.57182 | -49.7056 |
| Awa-Guajá | -3.33685 | -46.4538 |
| Kayapó/Xikrin | -4.59294 | -51.5387 |
| Zo’é | -0.12834 | -55.7658 |
| Wayãpy | 1.209187 | -52.7701 |
| Munduruku | -6.4378 | -57.9655 |
| Caripuna | 3.559476 | -51.4918 |
